# Marine Collagen and Chitin: Promising Applications in Interdisciplinary Fields

**DOI:** 10.3390/md22090379

**Published:** 2024-08-23

**Authors:** Azizur Rahman

**Affiliations:** 1 Centre for Climate Change Research, University of Toronto, ONRamp at UTE, Toronto, ON M5G 0C6, Canada; aziz@climatechangeresearch.ca or mazizur.rahman@utoronto.ca; 2 A.R. Environmental Solutions, ICUBE-University of Toronto, Mississauga, ON L5L 1C6, Canada; 3 AR Biotech Canada, Toronto, ON M2H 3P8, Canada

Marine collagen and chitin derived from marine organisms are gaining significant attention for their diverse applications across various fields. This Editorial explores the potential of these biopolymers in different interdisciplinary applications. Chitin and that enzymatically deacetylated to chitosan have many applications, including in the medical, environmental, and agricultural sectors [1,2,3,4,5,6]. Likewise, nature is a source of significant quantities of collagen, especially in marine organisms. Marine-based collagen contributes greatly to biotechnology products and medical applications [7,8,9,10,11]. Considering the importance of these two biopolymers and their multidisciplinary applications, I was interested in editing this Special Issue.

Highlighted below are the interdisciplinary applications of collagen and chitin:

## 1. Biomedical Applications

### 1.1. Wound Healing

Collagen: Promotes cellular regeneration and provides a scaffold for new tissue formation [9,10].

Chitin: Exhibits antibacterial properties, reducing infection risks and enhancing healing.

### 1.2. Drug Delivery Systems

Collagen: Used for targeted drug delivery due to its biocompatibility and ability to be engineered into nanoparticles.

Chitin: Chitosan, a derivative of chitin, forms hydrogels and nanoparticles, improving the controlled release of drugs.

### 1.3. Tissue Engineering

Collagen: Supports the growth of various cell types and can be used to engineer tissues such as skin, bone, and cartilage.

Chitin: Provides structural integrity and can be combined with other materials for scaffolding in tissue engineering.

## 2. Cosmetic Industry

### 2.1. Anti-Aging Products

Collagen: Hydrates the skin, improves elasticity, and reduces wrinkles.

Chitin: Enhances moisture retention and provides a protective barrier.

### 2.2. Hair and Nail Care

Collagen: Strengthens hair and nails, promoting growth and resilience.

Chitin: Adds volume and shine to hair and protects nails from damage.

## 3. Environmental Applications

### 3.1. Biodegradable Materials

Collagen: Used to produce biodegradable films and packaging materials.

Chitin: Forms biodegradable plastics and can be used in the production of eco-friendly materials.

### 3.2. Waste Water Treatment

Collagen: Acts as a flocculant, helping to aggregate and remove contaminants.

Chitin: Chitosan efficiently absorbs heavy metals and organic pollutants from water.

## 4. Agricultural Sector

### 4.1. Soil Health

Collagen: Improves soil structure and fertility when used as an amendment.

Chitin: Acts as a biopesticide and enhances the growth of beneficial microorganisms in the soil.

### 4.2. Animal Feed

Collagen: Enhances the nutritional quality of animal feed, promoting better growth and health.

Chitin: Improves gut health and immunity in livestock.

## 5. Food Industry

### 5.1. Functional Foods

Collagen: Used in supplements and health drinks for its joint and skin benefits.

Chitin: Acts as a dietary fiber, aiding in digestion and weight management.

### 5.2. Food Packaging

Collagen: Creates edible films that preserve food quality and extend shelf life.

Chitin: Chitosan coatings prevent microbial growth and spoilage.

This Special Issue, “**Collagen and Chitin from Marine Resources and Their Interdisciplinary Applications**”, contains 10 original and 3 review articles. An overview of the research results and reviews of the existing public literature by the authors is provided, which can help readers find appropriate articles in their field of interest. The contributions are listed in the List of Contributions.

Contribution 1 provides valuable information for the establishment of *Chondrosia reniformis* as an ecological and biomedically relevant source of collagen and is paramount for the future development of marine collagen-based biomaterials.

Contribution 2 investigates the effect of low-molecular-weight fish collagen (valine-glycine-proline-hydroxyproline-glycine-proline-alanine-glycine; LMWCP) on H_2_O_2_- or LPS-treated primary chondrocytes and monoiodoacetate (MIA)-induced osteoarthritis rat models.

In contribution 3, the authors report the carboxymethyl chitosan derivatives in bearing quinoline groups that showed remarkable antioxidant ability and weak cytotoxicity, highlighting their potential use in food and medical applications. The results indicate that most of the derivatives exhibited strong free radical scavenging ability while also showing low cytotoxicity.

In contribution 4, the authors extract chitosan from marine amphipods associated with aquaculture facilities and test its use in crop protection. This new chitosan valorizes aquaculture residues and has the potential to manage diseases in food security crops such as bananas. This is the first time that chitosan has been obtained from biofouling amphipods.

**Figure 1:**
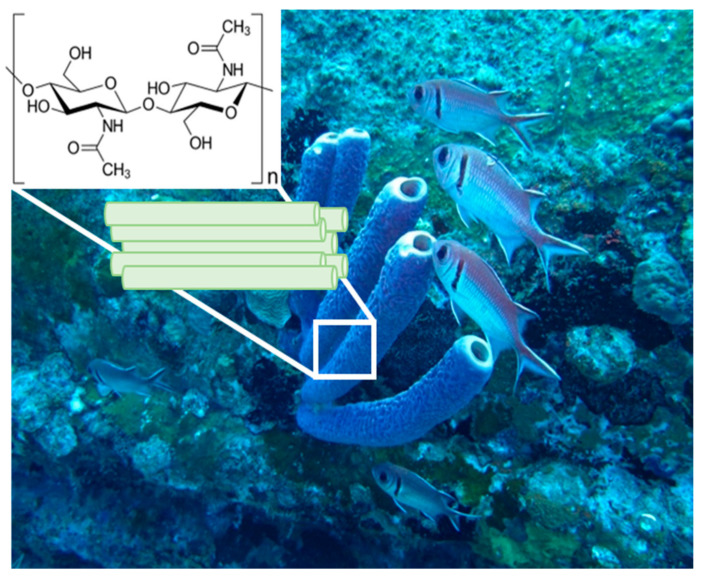
An underwater image of 30 cm long marine demosponges belonging to the Verongiida order in their original environment (photograph courtesy of Dr. V. Ivanenko).

In contribution 5, Ehrlich, H. and his team [12] report the mechanical properties of microfiber-based 3D chitinous scaffolds from selected Verongiida sponges. In this manuscript, the authors show that the scaffolds isolated from diverse representatives of cultivated marine demosponges, which belong to the Verongiida order (Figure 1), remain candidates with high potential in biomedicine and bioinspired materials science.

In contribution 6, the authors highlight the interaction between biomaterial spongin and iron ions in marine environments due to biocorrosion, which leads to the occurrence of the biomineral lepidocrocite. For this purpose, a biomimetic approach for the creation of a new lepidocrocite-containing 3D spongin scaffold using artificial seawater and iron powder under laboratory conditions at 24 °C is described for the first time.

In contribution 7, the authors demonstrate a holistic model for the chitinolytic machinery of *Jeongeupia* spp. based on cumulative data found by conducting proteomic and transcriptomic analyses. In this study, the authors identified the involvement of over 350 unique enzymes and 570 unique genes in the catabolic chitin response of a Gram-negative bacterium through three-way systems biology.

**Figure 2:**
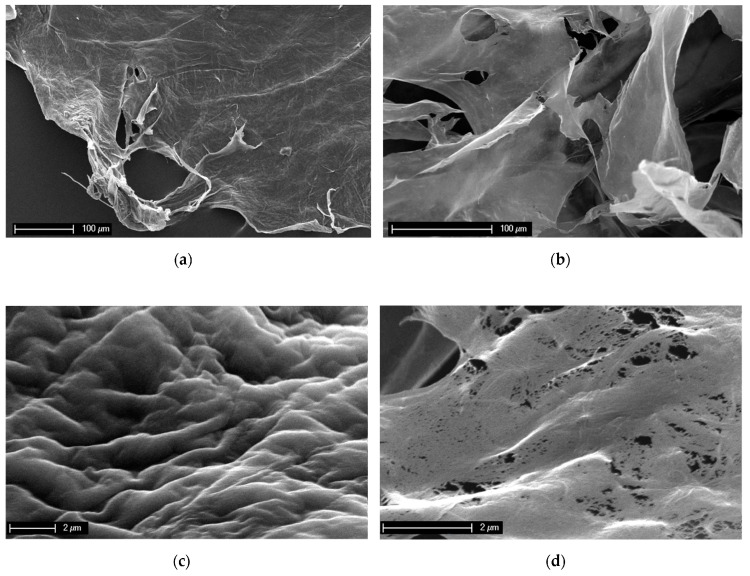
SEM images of the *A. aerophoba* chitin scaffold after NaOH treatment (**a**,**c**) and after dissolution in LiOH (**b**,**d**). The destruction of the structural integrity after insertion into LiOH solution at the micro-level is clearly visible.

Contribution 8 [13] shows a loss of structural integrity in 3D chitin scaffolds from a marine demosponge, *Aplysina aerophoba*, after treatment with LiOH. Figure 2 shows SEM images of the sponge chitin fibers isolated after NaOH treatment (a,c) and those after dissolution in LiOH solution (b,d). The difference in the structural integrity is clear. The surface of the NaOH-treated chitin scaffold is rough and monolithic (Figure 2a,c). The micrograph of the dissolved sponge chitin after freeze-drying presents a structure consisting of smooth layers (Figure 2b). A closer look at the disintegrated smooth surface reveals the presence of chitin nanofibers (Figure 2d).

Contribution 9 reports a thermostable type I collagen from a swim bladder of silver carp, *Hypophthalmichthys molitrix*. In this report, collagen from the swim bladder of silver carp and collagens from the swim bladders of grass carp, bovine pericardium, and mouse tail were extracted for comparison using the pepsin extraction method. SDS-PAGE, peptide maps, and amino acid composition identified the compositional differences in the isolated collagens. Their physicochemical properties were determined using ultraviolet–visible spectroscopic (UV) analysis, Fourier transform infrared spectroscopy (FTIR) analysis, and circular dichroism (CD) measurement. In addition, the thermal stability of the isolated collagens was confirmed by CD. Also, the fibril-forming and antioxidant capacities of the isolated collagens were investigated. These results indicate that the swim bladder of silver carp, *Hypophthalmichthys molitrix*, is a promising alternative source of mammalian collagen for pharmaceutical and biomedical applications.

Contribution 10 synthesizes chitin derivatives with propane sulfonated groups and demonstrates potential antioxidant and antifungal activity. The chitin and sulfonated chitin derivatives were tested in vitro for antioxidant and antifungal activity.

**Figure 3:**
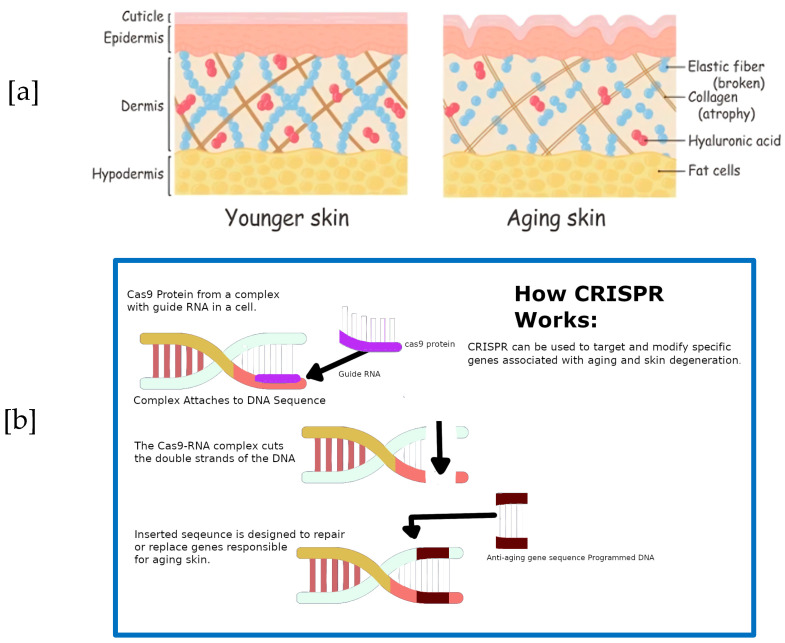
(**a**)**.** Illustration of the structural differences between younger and aging skin. In the dermis of young human skin, collagen fibrils are intact and normal in size (**left**), in contrast with reduced collagen fibrils in the dermis of aged human skin, which leads to a reduction in cell size (**right**). The aging skin on the right shows a reduction and fragmentation of collagen fibers, broken elastic fibers, and diminished Hyaluronic Acid (red dots), leading to thinner fat layers and an overall loss of structural integrity and elasticity. (**b**). Illustration of the CRISPR-Cas9 mechanism for skin regeneration. This graphic outlines the use of CRISPR-Cas9 technology for targeted gene editing in eukaryotic cells, specifically for skin regeneration. The process begins with the Cas9 protein forming a complex with a guide RNA that is complementary to a specific gene sequence associated with skin aging. This complex then locates and binds to the target DNA sequence, where Cas9 makes a precise cut. A new DNA sequence with the desired genetic information can then be inserted at the cut site for potential therapeutic purposes, such as reversing aging effects or repairing skin damage.

In contribution 11, Rahman et al. [14] report a scientific exploration for delaying skin aging by applying the therapeutic potential of marine collagen. Figure 3a shows how marine collagen proteins can delay skin aging. The authors also explore how CRISPR technology could be used to target and modify specific genes associated with aging and skin degeneration. An overview of this technology can be seen in Figure 3b.

In contribution 12, the authors review the function of chitosan in dental implantology. This review discusses chitosan as a biocompatible and bioactive material with many benefits in surgery, restorative dentistry, endodontics, prosthetics, orthodontics, and disinfection.

Contribution 13, a review, focuses on the scaffold polyvinyl alcohol (PVA)–chitosan (CS) and its multiple functions in tissue engineering and regenerative medicine applications. The authors also discuss the challenges and limitations associated with its use.

This Editorial’s concluding remarks are as follows:

The diverse applications of marine collagen and chitin across various fields highlight their interdisciplinary potential. The continued research and development of these biopolymers can lead to innovative solutions addressing current challenges in medicine, cosmetics, the environment, agriculture, and the food industry.

I want to thank the Editorial Board, Managing Editors and Editorial Assistant. I greatly appreciate the efforts provided by the authors who contributed their results to this Special Issue. I thank the reviewers who carefully evaluated the submitted manuscripts for their support.
